# Creation of artificial skyrmions and antiskyrmions by anisotropy engineering

**DOI:** 10.1038/srep31248

**Published:** 2016-08-10

**Authors:** S. Zhang, A. K. Petford-Long, C. Phatak

**Affiliations:** 1 Materials Science Division, Argonne National Laboratory, 9700 S. Cass Avenue, Argonne, IL 60439, USA; 2 Dept. of Materials Science and Engineering, Northwestern University, 2220 Campus Drive, Evanston, IL 60208, USA

## Abstract

Topologically non-trivial spin textures form a fundamental paradigm in solid-state physics and present unique opportunities to explore exciting phenomena such as the topological Hall effect. One such texture is a skyrmion, in which the spins can be mapped to point in all directions wrapping around a sphere. Understanding the formation of these spin textures, and their energetic stability, is crucial in order to control their behavior. In this work, we report on controlling the perpendicular anisotropy of continuous Co/Pt multilayer films with ion irradiation to form unique spin configurations of artificial skyrmions and antiskyrmions that are stabilized by their demagnetization energy. We elucidate their behavior using aberration-corrected Lorentz transmission electron microscopy. We also discuss the energetic stability of these structures studied through *in-situ* magnetizing experiments performed at room temperature, combined with micromagnetic simulations that successfully reproduce the spin textures and behavior. This research offers new opportunities towards creation of artificial skyrmion or antiskyrmion lattices that can be used to investigate not only fundamental properties of their interaction with electron currents but also technological applications such as artificial magnonic crystals.

In this ‘big data’ era, the quest for more energy-efficient information technology has encouraged research in nanomagnetic materials with a tunable magnetic domain behavior. One potential candidate for carrying information is a topologically non-trivial spin texture known as a skyrmion^1^,^2^,^3^,^4^, whose spins can be mapped to point in all of the directions wrapping a sphere, with the spins at the skyrmion core and outer edge both pointing out-of-plane but in opposite directions. One of the interests in these spin textures is the topological Hall effect^4^,^5^: when an electron current encounters a skyrmion, the electrons are deflected due to the emergent magnetic field of the skyrmion. Technologically, it has been predicted that skyrmions can be easily controlled using spin transfer-torque effects, thus requiring very low electric current to drive their motion^6^. In spite of the recent progress however there are still several limitations in our understanding of the fundamental physics of their behavior.

Topologically the spin structures can be characterized by a topological index, the winding number, which is defined by:


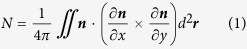


where ***n*** is the unit vector of the local magnetization^4^. For a skyrmion spin texture, the winding number, often referred to as the ‘skyrmion number’ is an integer, |*N*_*sk*_| ≥ 1. Note that the winding number of another non-trivial spin texture, the magnetic vortex state, is given by 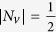. In addition to the skyrmion number *N*_*sk*_, the skyrmion spin structure can be characterized by the vorticity, *m*, and helicity, *γ*, which were defined by Nagaosa and Tokura^4^. For a given boundary condition at *r* = ∞, the vorticity directly corresponds to the skyrmion number, therefore spin structures with *m* = 1 are skyrmions, and those with *m* = −1 are antiskyrmions^7^. All the antiskyrmion structures are equivalent on rotation in the *x-y* plane. Similarly skyrmions with helicity *γ* = 0 or π, and with *γ* = ± π/2 are identified as Néel-type (hedgehog) and Bloch-type (spiral) skyrmions respectively. In bulk magnetic materials, these spin states typically arise from the bulk Dzyaloshinskii-Moriya (DM) interaction, and are most commonly seen in B20-type chiral single crystals that lack inversion symmetry, for example MnSi and FeGe, at very low temperature or near room temperature, in the presence of a magnetic field^1^,^2^,^8^,^9^. However, the weak DM interaction and the narrow region in the temperature-field phase diagram over which the skyrmions are stabilized, have limited the exploration of their properties. Skyrmions have also been observed in ferromagnetic thin films with perpendicular anisotropy as a result of the interfacial DM interaction, such as in Fe monolayers grown on Ir (111)^10^. Room temperature observation of micron-sized Néel-type skyrmion bubbles, which are stabilized by the interfacial DM interaction, has also been reported^11^,^12^,^13^,^14^.

An alternative approach that can stabilize the skyrmion spin texture relies on using dipolar interactions instead of DM interactions. The advantage of using dipolar interactions is that this approach does not require broken inversion symmetry; however, the size of the skyrmion structure is typically larger than those formed due to DM interactions. The dipolar interactions also result in the formation of skyrmions with both chiralities (positive and negative) in the same material, as opposed to skyrmions formed due to DM interactions^9^, which all have the same chirality. Dipolar interactions, and in particular the demagnetizing energy, have been used for the creation of artificial skyrmions^15^,^16^,^17^,^18^, which have, to date, been created by imprinting the magnetic vortex structure of circular discs fabricated on top of perpendicularly-magnetized thin films, e,g., Co discs on top of Ni(30 monolayers)/Cu(001)^19^. However, this approach introduces an extra layer of material, namely Co in the above example, which will affect the electrical and magnetic properties (such as the transport measurement) of the underlying skyrmions. Here we report on the creation of artificial skyrmions and antiskyrmions via localized ion irradiation of Co/Pt multilayers, to create circular regions with in-plane magnetization set into the surrounding perpendicularly-magnetized film. These artificial skyrmion and antiskyrmion spin textures are stabilized due to the dipolar interactions, i.e., the demagnetization energy, are stable at room temperature, and can be manipulated by an external magnetic field. By exploring the magnetization reversal process of the spin structures that are created by ion irradiation, we elucidate their energetic stability. This study opens new pathways to control the magnetic and electrical properties of skyrmions and antiskyrmions, such as transport measurements and the skyrmion spin-wave behavior for magnonic applications^20^. The potential impact of this work is that it enables local control and continuous tunability of magnetic anisotropy that can be tailored for desired technological applications.

## Results

### Ion irradiation effects on anisotropy

**Figure 1:**
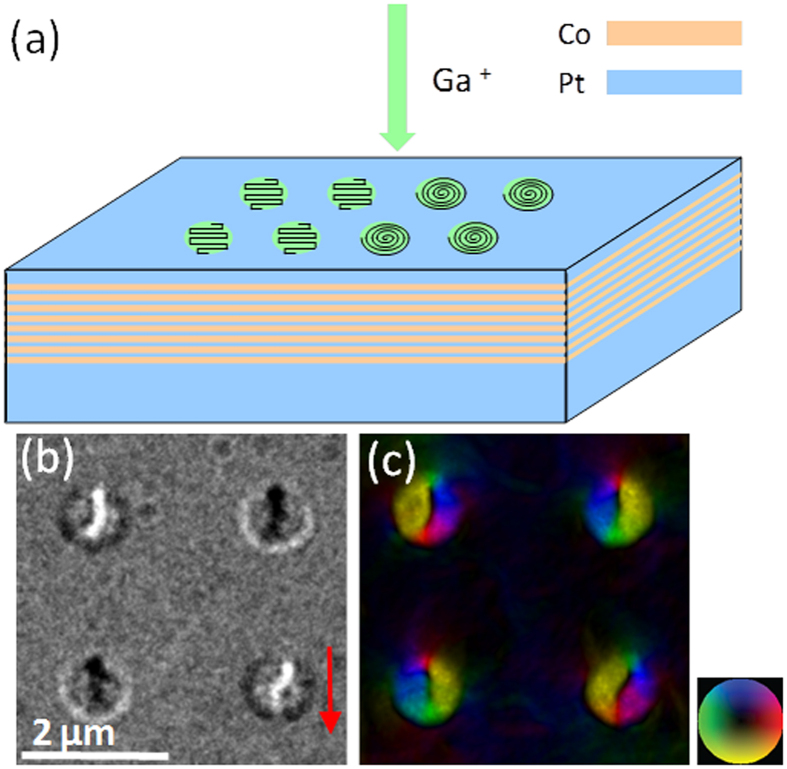
Schematic of ion-irradiated Co/Pt multilayer and LTEM image of circular raster-scanned irradiated regions. (**a**) Ga^+^ ion-irradiated Pt(10.0 nm)/[Co(0.3 nm)/Pt(1.0 nm)]_8_/Pt(2.0 nm) multilayer film. Circular regions with diameters of 300 nm to 1 μm were patterned using either a raster scan (left side) or a spiral scan (right side). (**b**) Under-focus LTEM image of raster-scan irradiated regions (patterning direction is shown by the red arrow). (**c**) Corresponding phase-reconstructed in-plane magnetic induction color map, showing an extended skyrmion core nucleated in each irradiated region, originated from the initial FIB patterning site (top of the circular region). The magnetization direction is indicated by the color wheel.

Co/Pt and Co/Pd thin-film magnetic multilayers have been intensively studied as they exhibit perpendicular magnetic anisotropy due to spin-orbit coupling effects at the interface^21^,^22^. By using asymmetric multilayers such as Ir/Co/Pt with broken inversion symmetry, it is possible to introduce an interfacial DM interaction^13^. Recent studies have shown that such multilayer systems offer thermal stability for skyrmion-like structures because they have a larger magnetic mass^23^. Ion irradiation of such multilayer films offers an additional way to tailor their domain behavior because under appropriate conditions, it has been shown to change the magnetization to lie in the plane of the film and to reduce the coercivity^24^,^25^,^26^,^27^,^28^,^29^,^30^,^31^. This is mainly as a result of intermixing at the interfaces leading to formation of a disordered mixed Co-Pt phase after irradiation^32^,^33^. Upon low ion dose irradiation, the surface roughness and topography of the irradiated films show little or no change as compared to the as-deposited multilayers^33^,^34^,^35^. In this work, we have used a focused ion beam (FIB) system with a Ga^+^ ion beam to irradiate circular regions with diameters from 1 μm down to 300 nm in Pt(10.0 nm)/[Co(0.3 nm)/Pt(1.0 nm)]_8_/Pt(2.0 nm) multilayers (shown in Fig. 1(a)). FIB irradiation enables choice of pattern and patterning direction, as well as the ability to define multiple milling locations to form skyrmion lattices^36^. One method of patterning is to use a raster scan, where the overall patterning direction is perpendicular to the raster direction. An under-focus Lorentz transmission electron microscope (LTEM)^37^ image of a circular region ion-irradiated using a raster scan is shown in Fig. 1 (b). LTEM is only sensitive to in-plane magnetization in the sample at normal incidence. The black or white circle surrounding each region represents a 90° domain wall between the in-plane magnetization inside the ion-irradiated region and the surrounding out-of-plane magnetization in the unirradiated Co/Pt multilayer film. The black or white line seen inside each circular region represents a 180° domain wall separating two domains with in-plane magnetization. A single domain wall is visible in each of the ion-irradiated regions. The corresponding in-plane magnetic induction color map of the same region as shown in Fig. 1(b), is shown in Fig. 1(c). The color map was obtained by phase reconstruction from a through-focal series of LTEM images, using a phase retrieval method based on the transport-of-intensity equation^38^. The colors correspond to the in-plane directions of magnetization as indicated by the associated color wheel. The black contrast outside the irradiated region, and at the domain wall, indicates an out-of-plane magnetization direction confirmed by tilting the sample. The color map shows a closure domain configuration around each wall, which can therefore be considered as an extended vortex core with one end close to the edge of the circular irradiated region. A striking feature of the magnetization configuration seen in Fig. 1(b) is the black or white domain wall contrast surrounding each irradiated region. The contrast of this domain wall is opposite to the contrast of the extended vortex core inside the irradiated region in each case. This circular domain wall has a unique character because it separates the out-of-plane magnetized material surrounding the circular irradiated region from the in-plane magnetization inside it. It thus forms an artificial 90° domain wall that is analogous to the domain surrounding a Bloch-type skyrmion, where the magnetization goes from out-of-plane in the surrounding material to in-plane inside the skyrmion, with the core again having an out-of-plane magnetization. Therefore, these irradiated regions can be recognized as deformed skyrmions.

The extended skyrmion cores in all of the circular regions are roughly aligned in the same direction, which corresponds to the patterning direction for the FIB irradiation (red arrow in Fig. 1(b)). Additionally, the extended core does not run across the entire width of an irradiated region but originates near one edge, which corresponds to the starting point for the ion-beam irradiation. Additional tests with different FIB patterning directions using raster scans confirm that the extended vortex cores are always aligned in the patterning direction and nucleate close to the starting point of the pattern (see supplementary information). This shows that there is an additional artificial anisotropy being introduced into the magnetic film that can be controlled by the ion beam patterning protocol.

### Creation of artificial skyrmions and antiskyrmions

**Figure 2:**
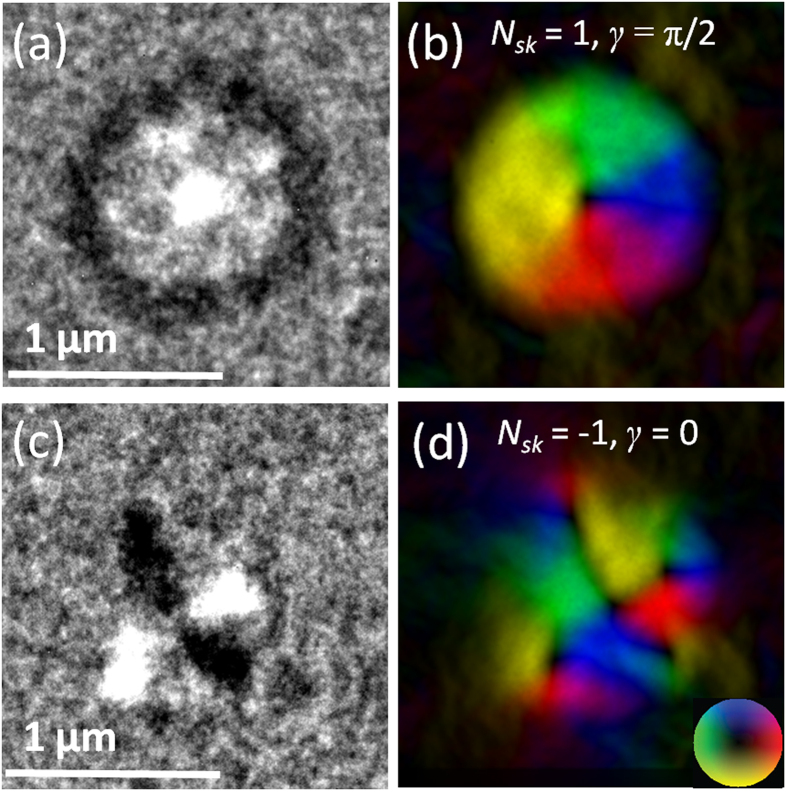
Under-focus LTEM images of circular ion-irradiated regions in a Co/Pt multilayer that form an artificial skyrmion and an artificial antiskyrmion. (**a**) Skyrmion and (**c**) antiskyrmion. The corresponding in-plane magnetic induction color maps are shown in (**b**,**d**) with in-plane magnetization directions represented by the color wheel. Black contrast in (**b**,**d**) indicates out-of-plane magnetization. Skyrmion number *N*_*sk*_ and helicity *γ* are indicated.

In order to control the asymmetric anisotropy induced by the raster scan, we used a circular FIB patterning scan, in which the beam moves in a spiral from center to edge (outward spiral), or from edge to center (inward spiral), in each of the circular regions. Figure 2 shows under-focus LTEM image of circular irradiated regions patterned using an outward spiral (Fig. 2(a)) and an inward spiral (Fig. 2(c)) together with the corresponding in-plane magnetic induction color maps (Fig. 2(b,d)) respectively (the corresponding magnetization vector maps are shown in the supplementary information). The images were recorded prior to being subjected to an external magnetic field. When patterning with an outward spiral, a single dot contrast was observed in the center of the irradiated region, representing a vortex configuration. Since Lorentz TEM can only measure the in-plane component of the magnetic induction, the spin structures we observed may seem similar to a vortex state. However, it should be noted that the irradiated regions discussed here differ from patterned discs containing magnetic vortices. The structures presented in this paper are surrounded by the pristine Co/Pt multilayer film whose magnetization points out-of-plane. Thus there is a continuous transition of magnetization from out-of-plane to in-plane and then back to out-of-plane as one goes from outside the irradiated region to the center of the region. Irradiation thus leads to circular regions with the spin texture of a Bloch-type ‘artificial’ skyrmion. The 90° domain wall surrounding each circular region again has opposite contrast to that of the core (see supplementary information for a schematic diagram to illustrate the 90^o^ domain wall), as seen for the raster scanned regions.

When patterning using an inward spiral, a completely different spin structure was observed (Fig. 2(c)), which consists of one black and one white domain wall intersecting near the center of the circular region. The in-plane magnetic induction color map shows that the spins point towards and away from the intersection along two orthogonal directions, indicating an antivortex structure^39^. However, since the magnetization surrounding the irradiated region and the magnetization at its core both point out-of-plane, seen from the black contrast in the phase-reconstructed magnetic induction map in Fig. 2(d), this irradiated region has the spin texture of an antiskyrmion. No domain wall contrast is observed around the edge of the antiskyrmion in Fig. 2(c), since the magnetization direction is segmented into four regions which lie between the skyrmions. Therefore the domain wall also gets segmented into four regions and as a result there isn’t sufficient contrast seen for the domain wall. This is dependent on the location of the four surrounding skyrmions and in fact in the in-plane field sweep of antiskyrmions (see the next section), we do see such segmented domain walls such as the white contrast around the edge. But instead, each antiskyrmion is associated with four small skyrmions near its edge, which have alternating chirality, and are formed to minimize the demagnetization energy. To the best of our knowledge, this is the first time an artificial nanoscale antiskyrmion has been realized by experiment, and indeed it is only recently that there has been observation of an antiskyrmion spin structure in bulk MnGe, and only at low temperature (5 K) under a 2.4 T applied perpendicular field^40^.

In order to create a skyrmion or antiskyrmion with the spins at the core pointing in the opposite direction to those outside the irradiated region, a magnetic field can be applied along the out-of-plane direction, similar to the approach used by Sun *et al*.^15^. Since the saturation field of our Co/Pt multilayer film is around 200 Oe and the skyrmion core has a switching field higher than 600 Oe (see supplementary information for out-of-plane magnetizing experiments), we can first apply a field of 2000 Oe in one out-of-plane direction to fully magnetize both the multilayer film and the irradiated regions, and then apply a reverse field of ~200 Oe to set the magnetization of the multilayer film in the opposite direction while leaving the cores unchanged. With this field treatment we propose a protocol to achieve skyrmions with cores pointing in the opposite direction to the surrounding unirradiated multilayer film.

We can understand how the skyrmion or anti-skyrmion spin textures are created via ion irradiation by understanding the patterning sequence used in the FIB. For the raster scan, the beam moves in the *x-y* direction starting at an initial point on the edge and then moving back and forwards towards the end point. As the raster scan progresses, the region with in-plane magnetization gradually increases. In order to reduce the magnetostatic energy, the region forms a closure domain around a domain wall. The irregular shape does not support the formation of a circular vortex state. Additionally, at the initial irradiation point the ion beam sits for a slightly longer time before the scan commences, resulting in slight overexposure to the Ga^+^ beam at that point. This modifies the local energy landscape and acts as a pinning site for the extended vortex core. After irradiation, even though the entire circular region has in-plane magnetization, except at the vortex core position, as a result of the exchange energy, there is a significant energy cost in unpinning the extended vortex core that has formed, resulting in the presence of domain walls in the raster-scanned regions.

For the spiral scans however, the beam moves continuously in the *r-θ* direction to form the spiral. For the inside-out spiral scan a circular in-plane magnetized region is created after irradiation, with a small region at the center that has an even lower perpendicular anisotropy as a result of this being the initial ion beam position. This supports the formation of a vortex state due to shape anisotropy. This circular vortex state grows until the entire region has been irradiated, resulting in a skyrmion. On the other hand, for the outside-in spiral scan, overexposure at the initial irradiation point leads to a point at the edge of the circular region with lowest anisotropy. As the ion beam progresses around the spiral scan, an annular ring of in-plane magnetization is initially formed, which grows from the outside in as the ion irradiation progresses towards the center of the region. This results in the region having a completely different spin texture as compared to that created using the inside-out spiral scan.

**Figure 3:**
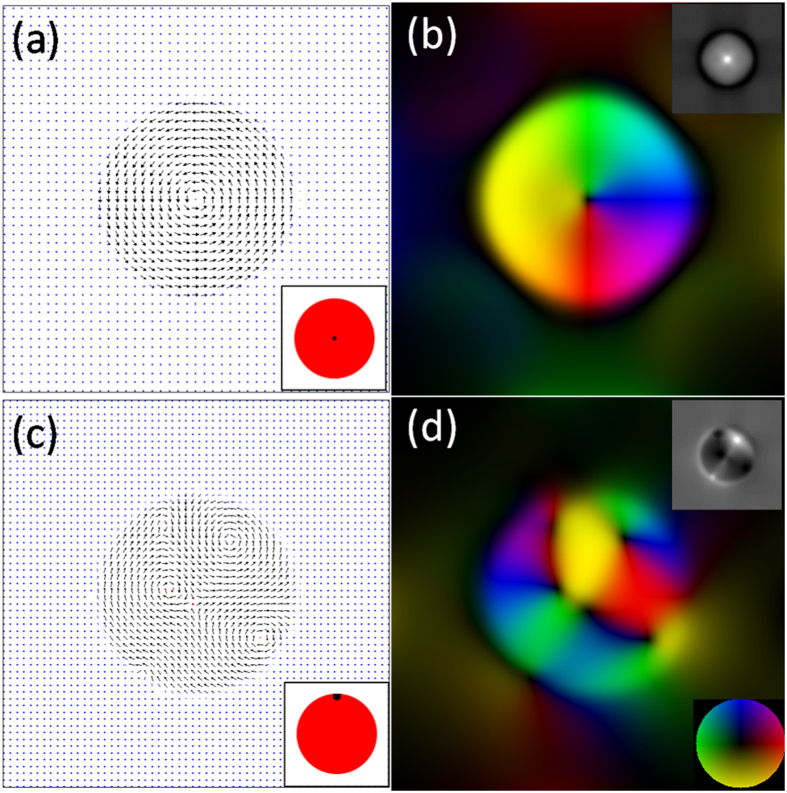
Micromagnetic simulations and corresponding in-plane magnetic induction color maps for 1 μm diameter circular ion-irradiated regions. (**a**,**b**) Skyrmion and (**c**,**d**) antiskyrmion. The blue and red in (**a**,**c**) indicate magnetization along +*z* and − *z* directions. Insets in (**a**,**c**) show the input configuration: white is the non-irradiated area, red is the irradiated region with reduced perpendicular anisotropy, and black shows a sub-region of further reduced perpendicular anisotropy to simulate the initial FIB-patterning site. In (**b**,**d**) the in-plane magnetization direction is represented by the color wheel and simulated LTEM images from the micromagnetic simulation results are shown as insets.

To investigate the mechanism by which the imprinted skyrmions or antiskyrmions are formed, we performed micromagnetic simulations by introducing a reduced perpendicular magnetic anisotropy in the circular ion-irradiated regions. For both raster-scanned and spiral-scanned regions, we observed that the skyrmion cores always nucleate at the location that was first exposed to ion irradiation, indicating an additional local modification of the intrinsic magnetic properties. This likely arises because of slight over-exposure to the Ga^+^ beam at the starting point, which modifies the local energy landscape. This effect was incorporated in the micromagnetic simulation by further local reduction of perpendicular anisotropy. The values of the anisotropy constant (K_1_) were taken to be one order smaller for ion-irradiated regions than for the non-irradiated Co/Pt multilayer based on results reported for a similar ion-irradiated Co/Pt multilayer system and the same 30 keV Ga^+^ ion dose by Rettner *et al*.^29^. Additionally, a further reduction in the anisotropy at the initial point of exposure to the FIB-irradiation was incorporated in the simulations. Chappert *et al*.^27^ reported that ion irradiation at a low dose (as used in our study) did not lead to an obvious reduction in magnetization, and we therefore used the same magnetization value throughout. Figure 3(a,b) show micromagnetic simulation result and corresponding in-plane magnetic induction color map for the remanent state of a 1 μm diameter circular region with further reduced anisotropy in the center, which successfully forms a skyrmion structure. Figure 3(c,d) show a metastable state for a circular low anisotropy region with further reduced anisotropy in a single region of diameter 100 nm at the edge, as shown in the inset schematic in Fig. 3(c). This metastable state is an antiskyrmion with four skyrmions surrounding it, and is a qualitative match to our experimental observation. This metastable state can remain for many steps during the simulation but eventually reverts to a circular spin structure. However, we believe that experimentally the metastable state is stabilized as a result of local pinning at the edges of the irradiated region due to the structural transition from well-ordered Co/Pt multilayer to disordered alloy. These simulations corroborate our hypothesis of further reduction in perpendicular anisotropy at the initial FIB patterning position as a result of a slight overexposure to the ion beam. The reduced perpendicular anisotropy K_1_ in the irradiated region can no longer support the out-of-plane magnetization, and therefore an in-plane chiral spin structure forms, as a result of the shape and aspect ratio of the irradiated region. However, near the core of this irradiated region, even though the out-of-plane anisotropy is low, the exchange energy dominates resulting in the magnetization pointing out of the film plane. We also carried out simulations with an applied field treatment as suggested earlier to obtain opposite magnetization direction for the core of the skyrmion or antiskyrmion and the surrounding Co/Pt film, which showed that the fields required for saturating the Co/Pt film and switching the core of the skyrmion differ by an order of magnitude.

The interfacial DM interaction in the unirradiated regions of the Co/Pt multilayer leads to the formation of Néel domain walls, which we observe using LTEM by tilting the sample (see supplementary material), similar as those reported by Benitez *et al*.^41^. However inside the ion-irradiated regions the multilayer has become intermixed, leading to formation of a disordered alloy, and a substantially reduced DM interaction. The DM interaction is negligible as indicated by the greatly reduced perpendicular anisotropy; therefore, artificial skyrmion and antiskyrmion spin textures can form in the irradiated regions, stabilized purely due to the dipolar interactions (demagnetization energy) resulting from their shape and size. We also observed skyrmions with both chiralities (positive and negative) present, which further indicates that the DM interaction does not play a strong role in their formation. Therefore, we treated the irradiated region as uniformly disordered region without any multilayers and did not include a DM interaction in our simulation.

### Stability and reproducibility

**Figure 4:**
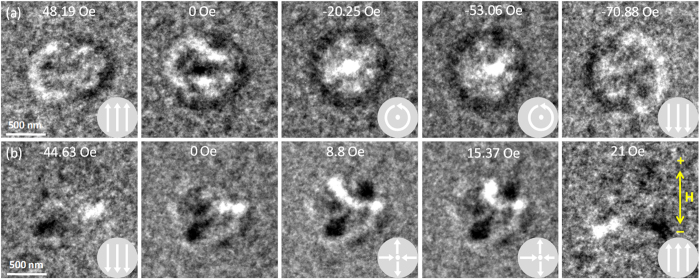
Under-focus LTEM images of circular ion-irradiated regions during *in-situ* magnetizing. The applied field value and direction are indicated. (**a**) Field applied from +750 Oe to −750 Oe to an artificial skyrmion. (**b**) Field applied from −750 Oe to +750 Oe to an artificial antiskyrmion. Left to right show selected LTEM images for representing states when the in-plane field was applied from one direction to the opposite. The ones at −53.06 Oe and 15.37 Oe show the change in core position for the skyrmion and antiskyrmion respectively. The direction of the applied field is indicated by the yellow arrow. Inset schematics depict the magnetization configuration in the circular region.

To further investigate the energetic stability of these artificial skyrmions and antiskyrmions, *in-situ* magnetizing experiments were performed using LTEM at room temperature, with an in-plane field sweeping between −750 Oe and +750 Oe. When the field was decreased from +750 Oe, some metastable states with complicated spin structures were observed, which ultimately resulted in the formation of a skyrmion or antiskyrmion (depending on the initial FIB patterning protocol) when the field was decreased to around −20 Oe, as shown by the under-focus LTEM images in Fig. 4. The applied field value and schematics of the magnetization configuration are indicated, as is the direction of the applied field (yellow arrow). As the field was decreased further, the skyrmion or antiskyrmion core moved towards the edge of the circular region in a direction that is roughly perpendicular to the applied field direction, which is similar to the behavior of a vortex or antivortex^42^,^43^,^44^,^45^. This phenomenon is not observed in mobile skyrmions since application of an in-plane field can lead to a change in either their size or their motion depending on the type of skyrmion. In our case however the locations of skyrmions are fixed, and so we only observe the motion of the core as a function of the applied field. The in-plane magnetization was aligned along the applied field direction at around −70 Oe. Reversing the applied field direction from −750 Oe to +750 Oe resulted in similar behavior, with a skyrmion or antiskyrmion appearing at around +6 Oe and the final state of magnetization being reached at around +50 Oe.

Repeating the *in-situ* magnetizing experiments multiple times on the artificial skyrmions, we observe that their helicity switches between −π/2 and +π/2. Repeating the experiments multiple times on the artificial antiskyrmions, we observe that their helicity can adopt any angle. There was no correlation observed between the helicity and the applied field direction, indicating that there is no other in-plane anisotropy introduced. This shows that the imprinted skyrmion or antiskyrmion state in the multilayer film is stabilized energetically due to dipolar fields even after the regions are fully magnetized. From the *in-situ* magnetizing study, we can also determine that these artificial spin textures are energetically stable within an applied field range of ±30 Oe. Thus, we have established the energetic stability criteria for these artificial skyrmions and antiskyrmions that could help in further analysis of their spin structure through other measurements such as the topological Hall effect.

## Discussion

**Figure 5:**
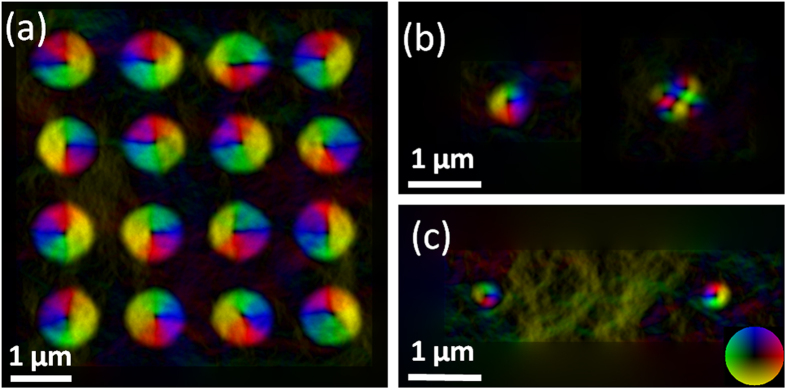
In-plane magnetic induction color maps of patterned arrays of artificial skyrmions and isolated skyrmions and antiskyrmions. (**a**) Patterned arrays of artificial skyrmions with 1 μm diameter and 500 nm separation. (**b**) One skyrmion and one antiskyrmion with 500 nm diameter. (**c**) Two skyrmions with 300 nm diameter. The in-plane magnetization directions are indicated by the color wheel.

FIB patterning offers the possibility to *locally* alter magnetic properties in a continuous multilayer film over nanometer length scales, enabling the creation of a variety of topological non-trivial spin structures such as skyrmions and antiskyrmions at any specific location. The skyrmions and antiskyrmions generated by ion irradiation can also easily be designed into lattice arrays with different geometries and lattice spacing, in contrast to the typically-observed hexagonal skyrmion lattice in chiral magnets^2^. Figure 5(a) shows an array of 1 μm diameter patterned skyrmions with 500 nm separation. The helicity is random, compared with skyrmions in chiral materials which prefer to have the same helicity in one domain^8^. Periodic artificial skyrmion lattices could contribute to a new form of 2D magnonic crystal that can be used to study skyrmion spin wave behavior in response to an applied spin-polarized current, as proposed by M. Krawczyk and D. Grundler^20^.

We also successfully created smaller skyrmions and antiskyrmions with sizes of 500 nm and 300 nm as shown in Fig. 5(b,c) respectively. The images were recorded after ion irradiation but prior to any field treatment. The high spatial resolution of the FIB beam (beam diameter about 5 nm when using 30 keV ions) makes it possible to achieve sub-100 nm sizes by precisely focusing the ion beam and tuning the ion dose. Additionally, since no extra layer materials are introduced on top of the film, we have the possibility to perform transport measurements on either isolated artificial skyrmions and antiskyrmions, or on periodic arrays. Transport has never been measured experimentally on antiskyrmions. With a low dose of 1.46 × 10^13^ ions/cm^2^, the total number of irradiated Ga^+^ ions is over two orders lower than the total Co atoms in the circular region, and the actual number of implanted Ga^+^ ions is lower than that as a result of back scattering and transmission of the 30 keV Ga^+^ ions into the substrate. We therefore think that the small amount of implanted Ga^+^ ions should have little impact on the transport properties. The fixed size and location of our patterned artificial skyrmions and antiskyrmions means that electron current flow cannot drive the entire spin structure to move, but their cores can still move inside the circular ion-irradiated regions as a result of the spin-transfer torque, similar to the skyrmion Hall effect^4^. LTEM combined with micromagnetic modeling will enable us to explore the quasi-static motion of the cores, leading to a better fundamental understanding of electron-skyrmion and electron-antiskyrmion interactions. Skyrmions written in precisely defined locations could also be useful for seeding such quasi-particles in spintronic devices such as racetrack memory.

In conclusion, we have demonstrated the formation of artificial skyrmions and antiskyrmions in ion-irradiated circular regions in Co/Pt multilayer films, without the need for additional layers of material. In the raster-scanned regions, extended skyrmion cores were observed that nucleated at the starting site of FIB patterning, which induced a further reduced perpendicular anisotropy at that site. Artificial skyrmions and antiskyrmions were successfully generated by patterning circular regions using a spiral scan with the ion beam writing from center-to-edge (outward) and from edge-to-center (inward) respectively. These topological non-trivial spin structures formed as a result of the dipolar interactions (demagnetization energy) are stable at room temperature and their spin structure can be manipulated by an external magnetic field. FIB patterning offers precise control of the size and location of each skyrmion or antiskyrmion, which can then be designed into arrays with different geometries and spacing. This study opens a new avenue to investigate the magnetic and electrical properties of skyrmions and antiskyrmions, such as the skyrmion Hall effect and magnonic crystal behavior. The method of anisotropy engineering also has the potential for further extension to local control of functional properties in superconducting and ferroelectric materials.

## Methods

### Sample preparation

Continuous Co/Pt multilayer films with the structure Pt(10.0 nm)/[Co(0.3 nm)/Pt(1.0 nm)]_8_/Pt(2.0 nm) were sputter-deposited at room temperature onto 2 mm × 2 mm TEM grids with 50 nm thick silicon nitride (SiN) membrane windows. Superconducting quantum interference device (SQUID) measurements were used to confirm the out-of-plane anisotropy of the multilayers with a coercivity of around 85 Oe and saturation field of around 200 Oe (see supplementary information). The saturation magnetization is 340 × 10^3^ A/m and the anisotropy constant is around 9.5 × 10^4^ J/m^3^ if assuming the [Co(0.3 nm)/Pt(1.0 nm)]_8_ as one material. An FEI Nova 600 NanoLab FIB system was used to irradiate circular regions with diameters of 300 nm to 1 μm in the Co/Pt multilayers with 30 kV Ga+ ions and an ion beam current of 9.7 pA. The ion dose was controlled by setting the dwell time as 100 ns with single pass, which corresponds to a dose of 1.46 × 10^13 ^ions/cm^2^. Two different scan types were used: raster scans and spiral scans. During patterning using a bitmap image, the beam moves in a raster scan, with the overall patterning direction perpendicular to the raster direction. During spiral patterning, the scan direction was circular with the ion beam moving in a continuous inward- or outward-spiral by choosing “Inner to Outer” or “Outer to Inner” in the circular patterning option of the FEI FIB system. Atomic force microscopy (AFM) measurement shows that there is no difference of topography and surface roughness between the ion-irradiated areas and the non-irradiated region.

### Magnetic domain observation

The ion-irradiated Co/Pt multilayer films were studied using Lorentz transmission electron microscopy (LTEM) in a dedicated JEOL 2100F instrument with a spherical aberration corrector, which enables imaging of magnetic structure at a spatial resolution as high as a few nanometers depending on the material and defocus. The out-of-focus Fresnel imaging mode was used to observe the domain structures of the irradiated regions with a defocus value of 7.23 mm. The spatial resolution for imaging the magnetic induction in the ion-irradiated Co/Pt multilayer discussed here is about 30 nm for this defocus value. In-plane magnetic induction maps were then reconstructed from a through-focus series of images using a phase-retrieval method based on the transport-of-intensity equation (TIE) to determine the magnetization configuration^38^. *In-situ* LTEM magnetizing experiments were performed using a Hummingbird Scientific magnetizing holder which has built-in electromagnetic coils that can apply a uniform in-plane magnetic field up to ±750 Oe with smallest step size of 1 Oe. No out-of-plane field is applied by the holder. Magnetizing experiments with an out-of-plane field, as described in the supplementary material, were performed in a FEI Tecnai F20ST TEM/STEM using Lorentz mode, and the field was applied by adjusting the objective lens current.

### Micromagnetic simulation

The OOMMF (Object Oriented MicroMagnetic Framework) package was used to perform simulations of the ion-irradiated regions. The model contains three regions with different perpendicular anisotropy values for the non-irradiated and irradiated Co/Pt multilayer film and the starting sites of the FIB patterning. Bitmap images were used as the input for these regions (shown as insets in Fig. 3(a,c)): the white regions are the non-irradiated Co/Pt multilayer film with anisotropy constant K_1_ = 1 × 10^5^ J/m^3^ along the *z*-direction as from the SQUID measurement; the red regions are 1 μm diameter ion-irradiated regions with K_1_ = 1 × 10^4 ^J/m^3^ along the *z*-direction; and the black region of 100 nm diameter are the initial FIB patterning sites with K_1_ = 1 × 10^2^ J/m^3^ along the *z*-direction. The film thickness is 10 nm. The saturation magnetization M_S_ = 340 × 10^3^ A/m and the exchange constant A = 1.5 × 10^−11^ J/m for all the regions. The cell size is 5 × 5 × 5 nm^3^. The initial magnetic state of the ion-irradiated circular region was set as random and the magnetization was relaxed by using energy minimization. The output magnetization from OOMMF simulations is used to calculate the phase shift of the electrons in the LTEM experiments using the Mansuripur algorithm^46^. The through-focus series images are then simulated using the calculated phase shift and the transfer function of the microscope with the following parameters: voltage = 200 kV, spherical aberration = 500 μm, and defocus = 5 μm^47^ which is enough to generate a clear LTEM image, since there is no grain structure in the simulations in contrast to what is observed experimentally.

## Additional Information

**How to cite this article**: Zhang, S. *et al*. Creation of artificial skyrmions and antiskyrmions by anisotropy engineering. *Sci. Rep*. **6**, 31248; doi: 10.1038/srep31248 (2016).

## Supplementary Material

Supplementary Information
