# Supplementary material for: Creation of artificial skyrmions and antiskyrmions by anisotropy engineering
Source: Sci Rep. 2016 Aug 10;6:31248. doi: 10.1038/srep31248 (PMC4978955; doi:10.1038/srep31248)
Supplement: Supplementary Information [file srep31248-s1.pdf]

Supplementary materials: Creation of artificial skyrmions and antiskyrmions by anisotropy engineering

S. Zhang[1], A. K. Petford-Long[1,2] & C. Phatak[1]

[1]Materials Science Division, Argonne National Laboratory, 9700 S. Cass Avenue, Argonne, IL 60439, USA.

[2]Dept. of Materials Science and Engineering, Northwestern University, 2220 Campus Drive, Evanston, IL 60208, USA.

Correspondence and requests for materials should be addressed to C.P. (email: cd@anl.gov)

Supplementary Note 1

Superconducting quantum interference device (SQUID) measurements on as-grown Pt(10.0 nm)/[Co(0.3 nm)/Pt(1.0 nm)]$_8$ /Pt(2.0 nm) multilayer films are shown as Fig. S1, which confirm that the pristine sample has a major out-of-plane anisotropy. The out-of-plane coercivity is around 85 Oe and the saturation field is around 200 Oe. The saturation magnetization is 340 $\times 10^3$ A/m and the anisotropy constant is calculated as 9.5 $\times 10^4$ J/m^3 (assuming the [Co(0.3 nm)/Pt(1.0 nm)]$_8$ multilayer as a single material).

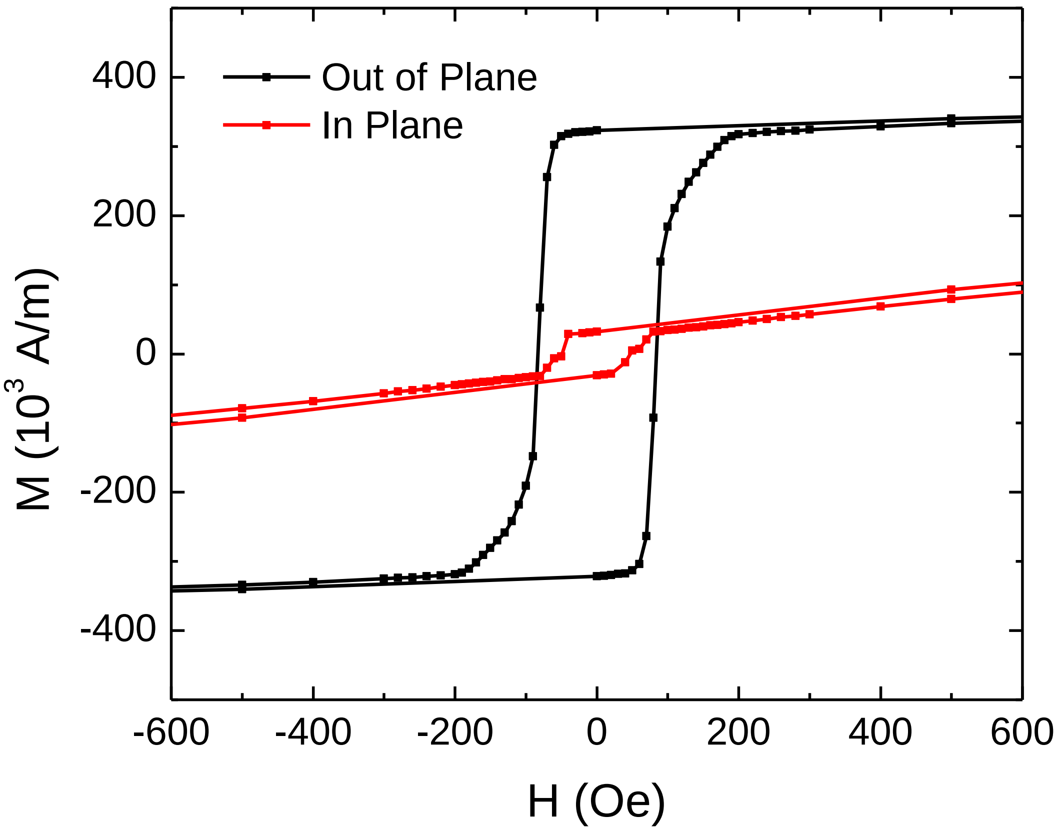

Fig. S1. SQUID data of as-grown Pt(10.0 nm)/[Co(0.3 nm)/Pt(1.0 nm)]$_8$ /Pt(2.0 nm) multilayer films.

Supplementary Note 2

Fig. S2 shows under-focus Lorentz TEM images of 1 μm diameter circular irradiated regions created using raster-scanning with a dose of 10^{14} ion/cm^2. The red arrows represent the ion beam patterning direction in each case. Almost all of the irradiated regions contain one deformed skyrmion. The deformed skyrmions are roughly aligned along the FIB patterning direction. In

addition, the magnetic domain wall contrast is strongest at the initial irradiation site, indicated by the yellow arrow, showing that the skyrmion was nucleated from that site, most likely as a result of slightly overexposure to the ion beam at the starting site. Fig. S3 shows under-focus Lorentz TEM images of spiral-scanned 1 μm diameter circular irradiated regions. Experiments carried out using a much longer dwell time (100 μs) enabled us to observe the ion beam motion using an oscilloscope and to show that the ion beam was moving in a continuous spiral no matter whether the starting point was in the center of the irradiated region or at the edge.

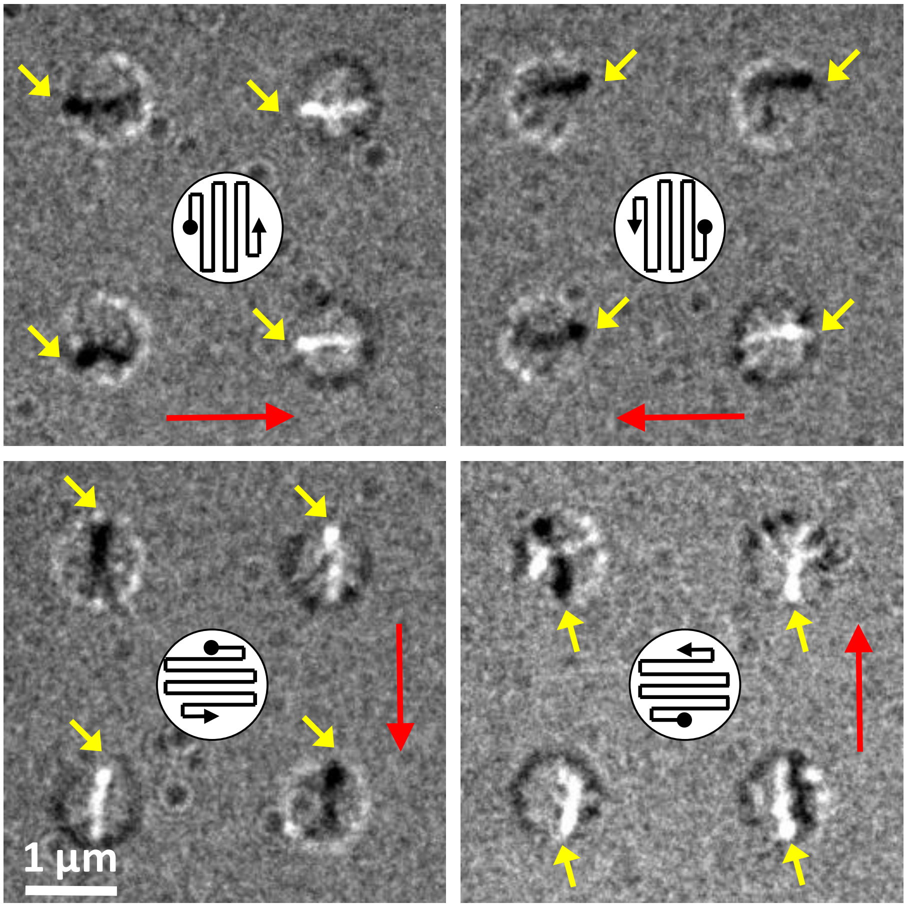

Fig. S2 Lorentz TEM images of circular regions that were ion irradiated with different FIB patterning directions, shown by the red arrows. The raster scan direction is sketched with a dot representing the starting point. The deformed skyrmions are nucleated close to the initial irradiation point (indicated by a yellow arrow), and are aligned roughly along the patterning direction.

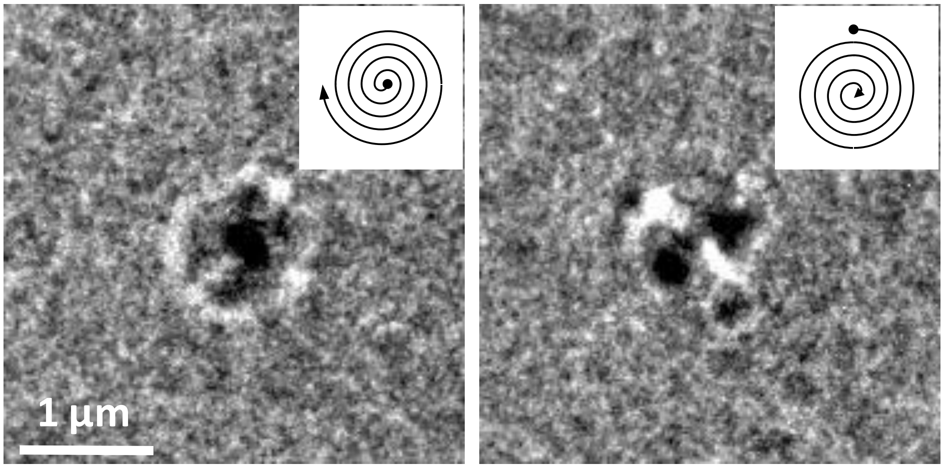

Fig. S3 Lorentz TEM images of ion irradiated circular regions with outward (left) and inward (right) spiral scan. Sketches of the beam scanning direction are shown as inset with a dot representing the starting point.

Supplementary Note 3

We also performed in-situ magnetizing experiments with an out-of-plane field. The field was applied by adjusting the objective lens current of the TEM. By tilting the sample, we can observe the domain wall structures in the unirradiated Co/Pt multilayer films, which are Néel type walls and similar as those reported by Benitez *et al.* in Ref. 41. As shown in Fig. S4, the unirradiated Co/Pt multilayer film was almost fully magnetized along the applied field direction at a field of 164 Oe, which is consistent with the saturation field of 200 Oe from the SQUID data seen in Fig. S1. In-situ magnetizing experiments with an out-of-plane field were also performed on artificial skyrmion and antiskyrmion structures, as shown in Fig. S5. The sample was not tilted for this measurement. The skyrmion and antiskyrmion spin structures started to change only for an applied field higher than 600 Oe, indicating that the switching fields of the cores is at least this value and is substantially higher than the saturation field of the surrounding multilayer film. The saturation field for the skyrmions and antiskyrmions is about 1200 Oe. Therefore, we can achieve skyrmions with cores whose magnetization points in the opposite direction to the surrounding unirradiated film by a field treatment, as proposed in the manuscript.

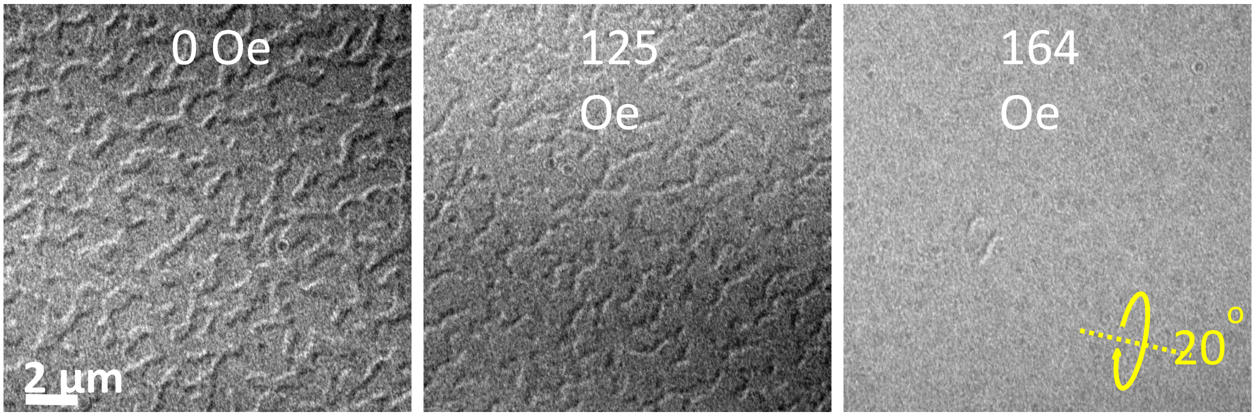

Fig. S4 Lorentz TEM images of unirradiated Co/Pt multilayer film for an out-of-plane applied field. The sample is tilted by 20 °in order to observe the Néel-type domain walls. These domain walls are induced by an interfacial DM interaction and a dipolar interaction. The out-of-plane component of the applied field value is shown in each figure.

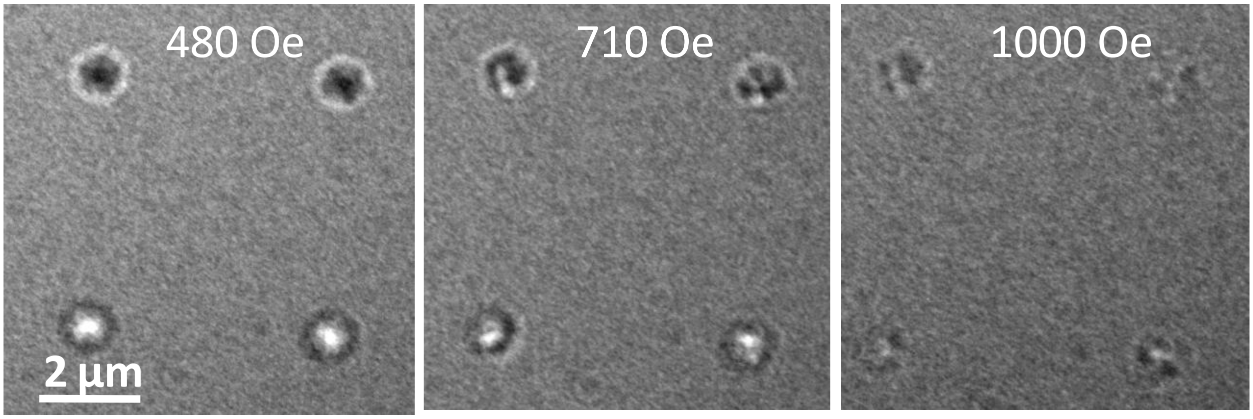

Fig. S5 Lorentz TEM images of circular regions ion-irradiated with an outward spiral, for various values of out-of-plane applied magnetic field. The in-plane circular spin structure begins to change when the out-of-plane field was increased to above 600 Oe, indicating that the skyrmion core has a switching field of at least 600 Oe. The saturation field is around1200 Oe.

Supplementary Note 4

To help identify the magnetization directions in the skyrmion and antiskyrmion structures, Fig. S6 shows the magnetization vector maps from the same phase reconstruction process of Fig. 2(b) and 2(d) in the main article respectively.

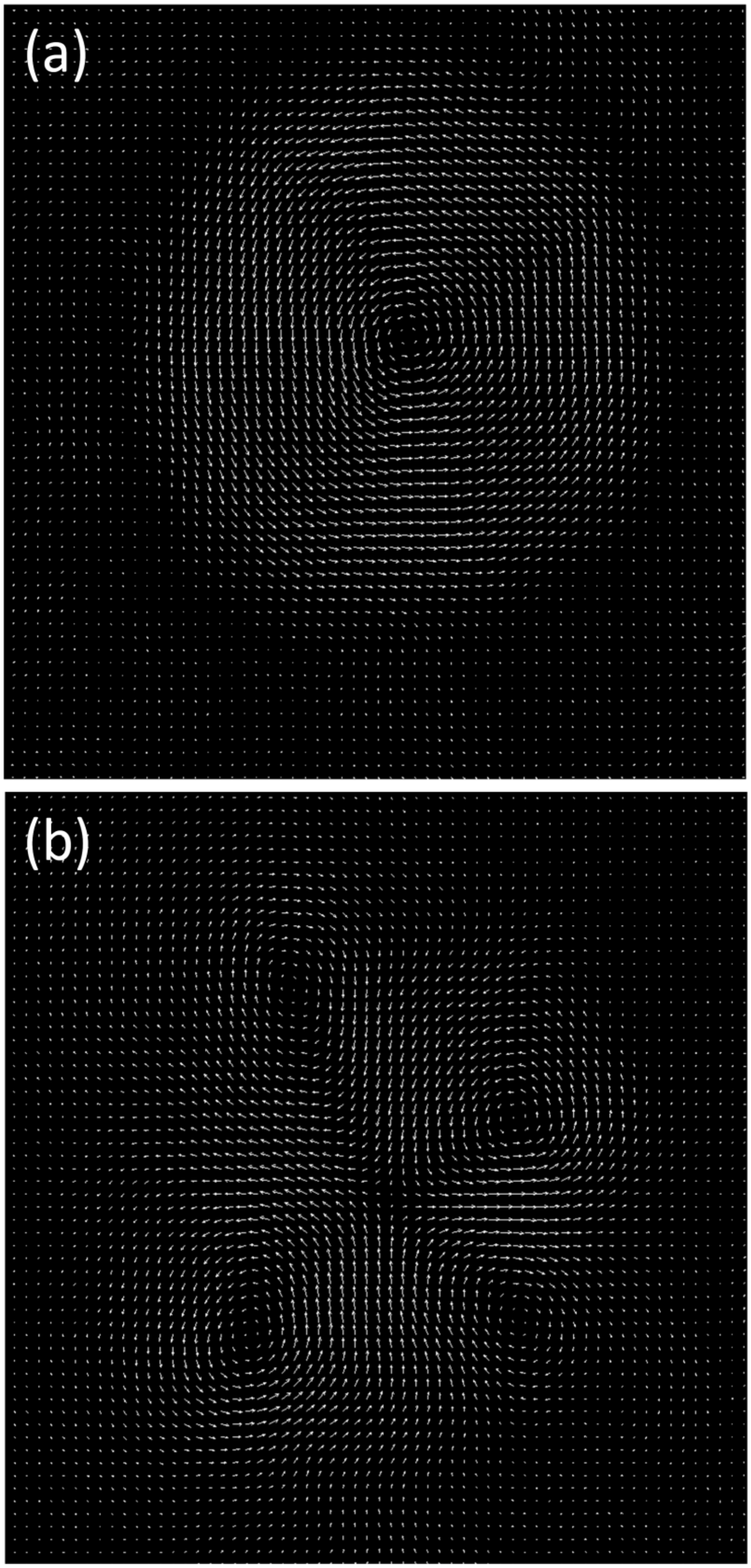

Fig. S6 Magnetization vector maps from the same phase reconstruction process of Fig. 2(b) and 2(d).

Supplementary Note 5

Fig. S7 shows a schematic diagram to illustrate the 90° domain wall in Fig. 2(a) of the main article.

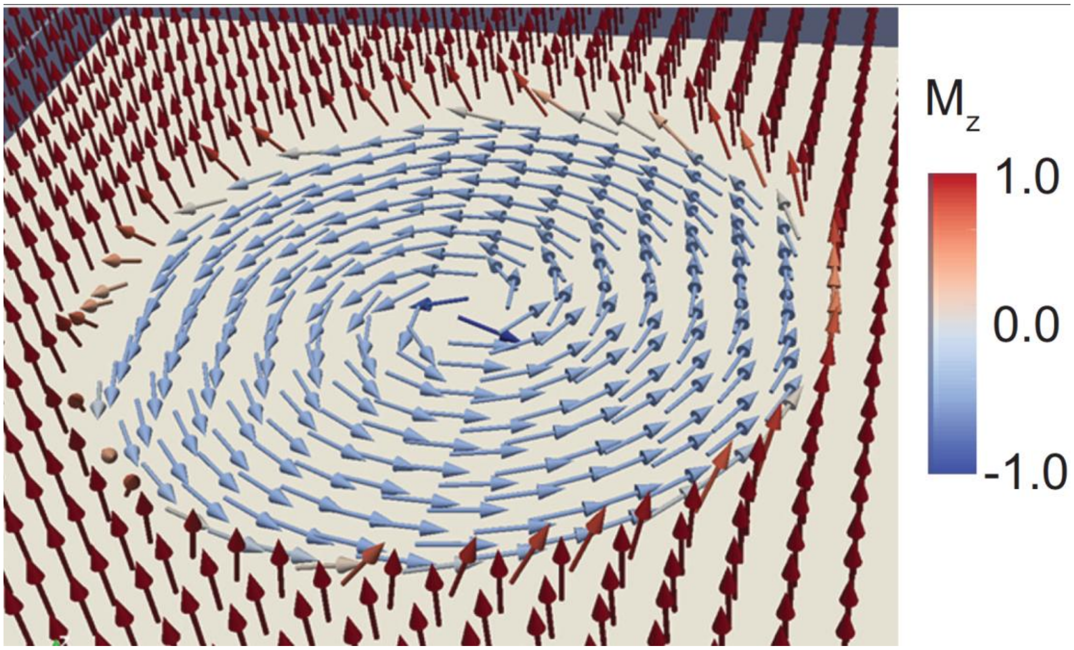

Fig. S7 A 3D schematic diagram of magnetization vectors showing the 90° domain wall at the edge of a Skyrmion. Red and blue colors represent the out-of-plane component of the vectors pointing out-of and into the plane of the paper respectively.
